# Bidirectional relationship between C-reactive protein and depressive symptoms considering cumulative effect among Chinese middle-aged and older adults

**DOI:** 10.3389/fpsyt.2024.1319682

**Published:** 2024-06-14

**Authors:** Ningxuan Zhao, Lin Jiang, Meijing Hu, Baiyang Zhang, Yidie Lin, Qiang Yao, Jingjing Hao, Cairong Zhu

**Affiliations:** Department of Epidemiology and Health Statistics, West China School of Public Health and West China Fourth Hospital, Sichuan University, Chengdu, China

**Keywords:** C-reactive protein, depressive symptoms, bi-directional relationships, population based, cumulative effect

## Abstract

**Introduction:**

Research examining the bidirectional relationship between C-reactive protein (CRP) and depressive symptoms, while accounting for cumulative effect of repeated episodes of CRP or depressive symptoms, is currently deficient in non-Western populations.

**Methods:**

A nationally representative population-based cohort data from the Chinese Health and Retirement Longitudinal Study (CHARLS) was utilized. In bi-directional analysis, we considered both single determinations and two successive determinations of CRP or depressive symptoms. Multivariate logistic regression assessed the association between elevated CRP levels at baseline or repeated episodes of CRP elevations over two successive determinations and subsequent elevated depressive symptoms, and vice versa.

**Results:**

Although single determinations of CRP or depressive symptoms yielded non-significant results in both directions, full multivariate models, adjusting for baseline depressive symptoms, socio-demographic characteristics, health-related behaviors, metabolic measures, and health status, revealed a significantly positive association based on two successive determinations of CRP or depressive symptoms. This significant association was observed between cumulative effects of sustained CRP elevations over two successive determinations (2 vs. 0) and subsequent elevated depressive symptoms (OR=1.58; 95% CI: 1.15 to 2.17) and between cumulative effect of repeated episodes of depression (2 vs. 0) and later elevated CRP (OR=1.26; 95% CI: 1.02 to 1.56). Furthermore, sex-stratified analyses confirmed the robustness of these relationships.

**Conclusion:**

There are bidirectional associations between depressive symptoms and CRP, driven by the cumulative effect of repeated episodes of CRP or depressive symptoms among middle-aged and older Chinese adults. These findings hold significant clinical implications, highlighting the potential of both anti-inflammatory and anti-depression approaches.

## 1 Introduction

Depression, particularly in rapidly aging societies, has become a burgeoning worldwide public health issue (1). China, as the world’s most populous nation, is currently experiencing a profound stage of rapid population aging (2). Based on a recent meta-analysis, depressive symptoms among older adults in China had an overall prevalence of 20%, imposing a substantial economic burden (3). Furthermore, depression has adverse effects on the standard of living among the elderly and was linked to a higher likelihood of mortality (4). Nevertheless, the pathophysiological mechanisms underlying the initiation and development of depression have remained elusive.

The “inflammation hypothesis” of depression, which emphasizes the role of psycho-neuroimmunological dysfunctions, has received mounting attention in recent research (5–7). The brain’s inflammatory responses may become impaired with age, potentially affecting the emergence of depressive syndromes (7). C-reactive protein (CRP), a commonly used indicator of inflammation, is increasingly recognized for its involvement in the pathophysiology of depression (8).

Despite extensive literature examining the association between CRP and subsequent depressive symptoms among older adults, the findings remain inconclusive. Some studies have indicated a positive link between CRP levels and later development of depressive symptoms (9–12), while others demonstrated null association (13–15). There are differences in the study design, the characteristics of the sample, the assessment instruments for depression, and the confounding factors adjusted across previous studies, which may partially explain the discrepancies in the reported conclusions. The conflicting results, to some degree, also showed the complexity of the association between CRP and depressive symptoms (10, 16, 17). One potential reason behind the inconsistencies in the published findings may be that most studies were based on the assessment of CRP for one single time, whereas it may be more appropriate to focus on the cumulative effects of sustained CRP elevations. Au et al. reported non-significant findings regarding the relationship between CRP levels and subsequent depressive symptoms when relying solely on the single assessment of CRP (17). In contrast, within the same cohort, Bell et al. demonstrated an association between cumulative effect of sustained CRP elevations and future elevated depressive symptoms (18). Additionally, Sonsin-Diaz et al. identified that individuals with sustained CRP elevations over three successive determinations were at higher risks of developing depression (19). However, studies considering the cumulative effect of sustained CRP elevations over two (or more) successive determinations primarily focused on European and American populations, with limited supporting evidence from China. Qin et al. revealed CRP at baseline was not associated with later depression in total sample of a nationally representative Chinese population (16). Nevertheless, their study only utilized a single assessment of CRP which may result in some degree of misclassification within the sample. Hence, further validation evidence is needed to generalize the association between the cumulative effect of sustained CRP elevations over more successive determinations and subsequent depressive symptoms.

Conversely, the presence of depressive symptoms may lead to elevated CRP levels, thus further complicating this intricate association. Kop et al. proposed potential biological mechanisms, such as the overactivity of the hypothalamic-pituitary-adrenal (HPA) axis and dysfunction in the autonomic nervous system (ANS), to explore how depression might influence inflammatory responses (20). Additionally, well-established manifestations of depression, such as negative emotions and stress, have been shown to stimulate the production of pro-inflammatory cytokines (21, 22). However, the findings of prior studies investigating the association between depressive symptoms and CRP among older adults also remain inconclusive (23–25). Similarly, ignoring cumulative effect of repeated episodes of depression may also indicated non-significant results. Copeland et al. found that a history of multiple depressive episodes exhibited a stronger correlation with CRP levels compared to solely considering the current depressive status (23). Therefore, conducting bidirectional research that considers the cumulative effect of repeated episodes of CRP or depressive symptoms is essential for a comprehensive understanding of the underlying mechanisms linking CRP and depressive symptoms. Nonetheless, most current investigations into the bidirectional relationship have solely concentrated on single determination of CRP or depressive symptoms (17, 24, 26–28). Li et al. examined the bidirectional relationship between CRP and depressive symptoms in Chinese data set without considering the effects of multiple episodes, potentially failing to accurately capture long-term patterns (28). Currently, there is a dearth of evidence exploring the cumulative effect of repeated episodes of CRP or depressive symptoms simultaneously in both directions among the Chinese population.

Therefore, the objective of this research is to examine the correlation between depressive symptoms and CRP and determine (a) whether bidirectional associations exist between depressive symptoms and CRP and (b) whether cumulative effect of repeated episodes of CRP or depressive symptoms would influence the two-way relationship between depressive symptoms and CRP in a nationally representative sample of Chinese middle-aged and older adults.

## 2 Materials and methods

### 2.1 Participants and procedures

The Chinese Health and Retirement Longitudinal Study (CHARLS) is a prospective survey conducted by Peking University’s National School of Development. To ensure the representation, the multistage stratified probability sampling method was used. It encompasses a wide range of areas in China, including 28 provinces, 150 counties/districts, and 450 villages/communities with the objective of supporting scientific research assistance for the population aged 45 years and older in China. Baseline questionnaire data were collected in 2011y through face-to-face computer-assisted personal interviews, and respondents were subsequently followed up in 2013y, 2015y, and 2018y. Baseline blood data were collected in 2011, while follow-up assessments occurred in 2015y. Further information regarding the study can be found in previous publication (29).

The current analysis focused on 11,847 individuals who accepted blood examinations at baseline. Participants were not included due to the following reasons: they were under the age of 45; they had serum hs-CRP levels≥ 10 mg/L and levels≤ 0.1 mg/L, as values exceeding this threshold were probably due to acute infection (30) and the lower limit of detection was 0.1 mg/L; they had self-reported memory-related disease and emotional, nervous, or psychiatric problems; or they had missing values for relevant covariates.

**Figure 1:**
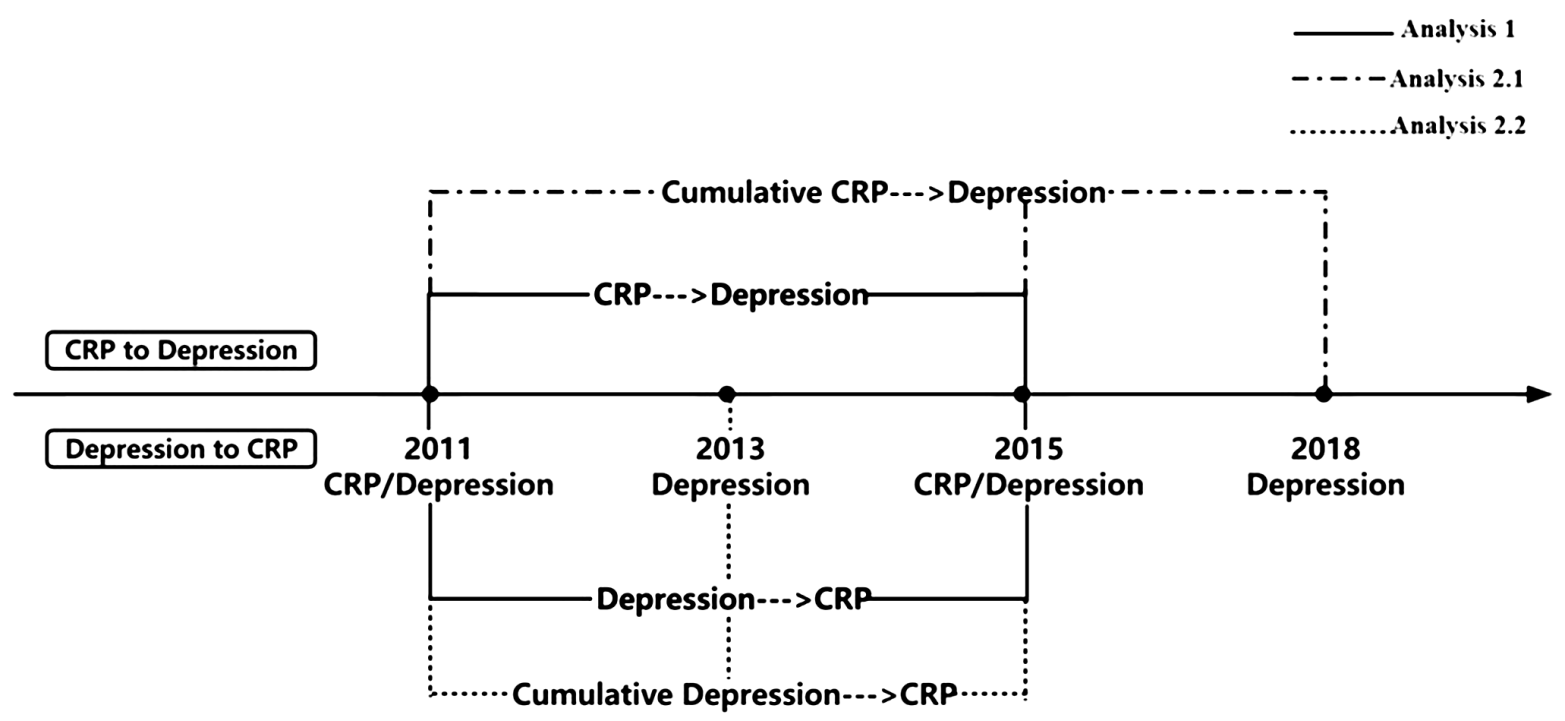
Outline of the prospective study design for relationship between depressive symptoms and CRP. CRP, C-reactive protein; Depression was assessed through the Center for Epidemiological Studies Depression 10 Scale.

**Figure 2:**
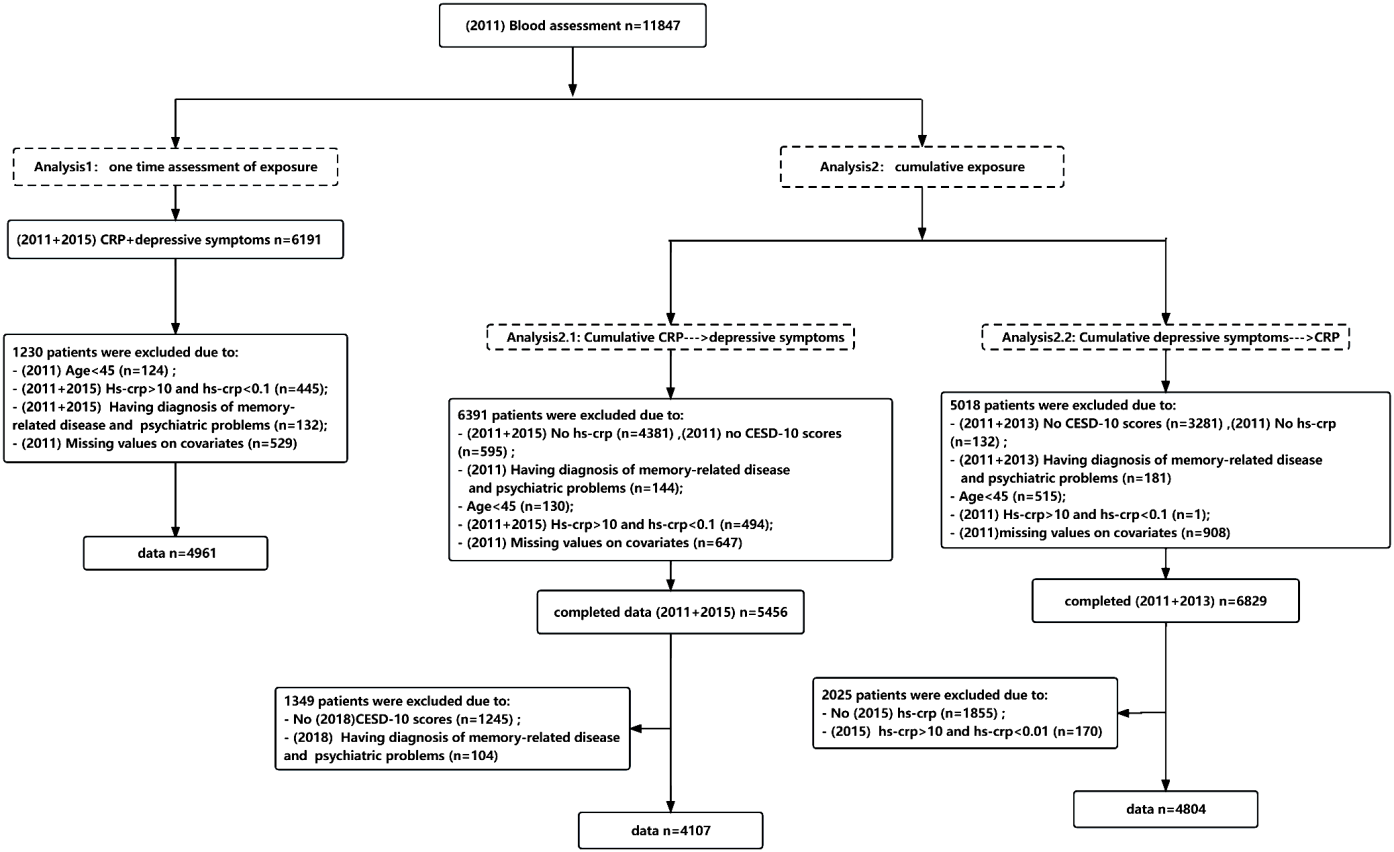
Flow chart of participant selection for this study.

The prospective study design is outlined in **Figure 1**. For the analysis of bidirectional associations based on only single assessment of CRP or depressive symptoms, we designated data from 2011y as Time 1 and data from 2015y as Time 2. Participants with available questionnaire and blood data for both years (2011y and 2015y) were included in Analysis 1 (N=6,191). When examining the cumulative effect of repeated episodes of CRP or depressive symptoms on the bidirectional relationship, we divided this analysis into two parts: 1) Cumulative effect of sustained CRP elevations to depressive symptoms; 2) Cumulative effect of repeated episodes of depression to CRP. In the first section, we used CRP data from 2011y and 2015y as the exposure, and we assessed depressive symptoms in 2018y as the outcome in Analysis 2.1 (N=4,107). Conversely, in the second section, the exposure was depressive symptoms from 2011y and 2013y, and the outcome was CRP in 2015y in Analysis 2.2 (N=4,804). Flowcharts illustrating the participant selection process are presented in **Figure 2**.

### 2.2 Measures

#### 2.2.1 Depressive symptoms

The assessment of depressive symptoms was conducted using the Center for Epidemiological Studies Depression Scale (CESD-10) that has good internal reliability and validity, appropriately reflecting individuals’ experience of depressive symptoms in the past week (31). The self-reported questionnaire consists of 10 items and are separated into “positive affect”, “depressive affect” and “somatic symptoms”. Each item was scored on a 4-point scale, with options ranging from “rarely or none of the time=0” to “all of the time=3”. The “positive affect” (item 5 and 8) is negatively worded and thus were reverse-scored. The total score, ranging from 0 to 30, was obtained by summing all item scores. Individuals were typically considered to have clinically relevant depression if they had a total score of 10 or more (32, 33). Cumulative effect of repeated episodes of depression over two successive determinations was determined based on the number of occasions when individuals scored ≥10 on the CESD-10 in either none, one, or both of the years 2011y and 2013y (range: 0–2).

#### 2.2.2 Blood and CRP data

Venous blood samples were obtained from each participant by medically-trained personnel affiliated with the Chinese Center for Control and Prevention (China CDC), adhering to a standardized protocol. The immunoturbidimetric assay was employed to quantify CRP levels in the plasma samples that had been frozen. The detection limit was 0.1–20mg/L and the coefficient of variation was 5.7% (29). At each visit, CRP was categorized into two groups: low (<3 mg/L) and elevated (≥3 mg/L) levels. Elevated CRP levels were classified as indicative of low-grade inflammation, a definition consistent with previous studies (17, 19). Cumulative effect of sustained CRP elevations over two successive determinations was determined based on the number of occasions when individuals level of CRP ≥3 during either none, one, or both of the years 2011y and 2015y (range: 0–2).

#### 2.2.3 Covariates

Socio-demographic characteristics (age, sex, residential area, education attainment, marital status), health related behaviors (tobacco smoking status, alcohol consumption, social activity), metabolic measures (body mass index [BMI], high density lipoprotein [HDL], triglycerides), and health status (hypertension, diabetes mellitus, cancer, Cardiovascular Disease [CVD], arthritic, asthma) at baseline were collected and included as covariates in the present analyses.

The classification of residential area included two categories, rural areas and urban areas. The data on educational attainment, marital status, tobacco smoking status, and alcohol consumption, etc. were collected by previously well-trained investigators in face-to-face interviews. Social activity, one of the variables, involves the assessment of a total of 10 activities individuals participated within 1 month before the survey. The ten activities included interaction with friends, taking part in community-related organization, attending educational or training courses, etc. The frequency of each activity was assigned a score as 0 (never), 1 (not regularly), 2 (almost every week), and 3 (almost daily). The frequency scores for the 10 activities were added up to generate the total score for social activity, which ranged from 0 to 30 and were categorized as 0, 1–2, and ≥3. Body mass index (BMI), derived from height and weight, was included in analyses as continuous variable. The enzymatic colorimetric test was employed by the Youanmen Center for Clinical Laboratory at Capital Medical University to analyze blood samples for high density lipoprotein (HDL) and triglycerides. Hypertension was defined as having an average systolic blood pressure (SBP) ≥140mmHg and/or an average diastolic blood pressure (DBP) ≥90 mmHg; or being prescribed antihypertensive medication; or reporting a medical history of diagnosed hypertension. Diabetes was defined as having a fasting plasma glucose level ≥126mg/dl (7.0mmol/L) and/or HbA1c ≥6.2mmol/L; or using any form of treatment for blood sugar control irrespective of fasting plasma glucose levels; or reporting a medical history of diagnosed diabetes. Self-reported doctor’s diagnosis of health conditions at baseline was recorded including cancer or malignant tumor (excluding minor skin cancers), CVD (including myocardial infarction, coronary heart disease, angina, stroke, and other heart problems), arthritis or rheumatoid arthritis, and asthma.

### 2.3 Statistical analysis

Our longitudinal analysis is divided into two parts: Analysis 1 explores the bidirectional associations using only single determination of CRP or depressive symptoms, while Analysis 2 is focusing on cumulative effect of repeated episodes of CRP or depressive symptoms. Baseline characteristics of study participants were described using Mean ± SD for continuous variables and frequency for categorical variables, stratified by CRP and depressive symptomatology groups.

We employed separate multivariate logistic regression models to investigate the bidirectional associations between CRP and depressive symptom based on both single determinations and two successive determinations of CRP or depressive symptoms. This allowed us to calculate odds ratios (ORs) along with their corresponding 95% confidence intervals (CIs). With reference to previous reports (17, 25, 34), five models had been constructed to explore the longitudinal and bidirectional relationship. For each regression model, the covariates were gradually added in the following order: Model 1 incorporated adjustment for the baseline levels of the outcome variable; Model 2 was Model 1 plus adjustment for sociodemographic data, including age, sex, education attainment, marital status, and residential area; Model 3 was Model 2 plus adjustment for health-related behaviors including tobacco smoking status, alcohol consumption, and social activity; Model 4 was Model 3 plus adjustment for metabolic factors, including BMI, HDL, and triglycerides; Model 5 was Model 4 plus adjustment for health conditions, including hypertension, diabetes mellitus, cancer, CVD, arthritic, asthma. Previous research had indicated gender differences in the relationship between CRP and depressive symptoms (35, 36). Therefore, we conducted sex-stratified analyses for each model.

Sensitivity analyses were conducted in three parts to assess result robustness. Firstly, Sensitivity Analyses 1 to 3 involved replicating the main analyses within a sub-cohort to assess the influence of excluding participants with elevated CRP or elevated depressive symptoms at baseline. Secondly, in the main analyses, while accounting for collinearity and the absence of CRP data in 2013y, we adjusted only for CRP levels or CESD-10 at baseline in Analysis 2.1 and Analysis 2.2. In Sensitivity Analysis 4, we accounted for the average of two observations of depressive symptoms in 2011y and 2015y when assessing the relationship between cumulative CRP and depressive symptoms. Finally, there are also differences in the calculation of cumulative effect of repeated episodes of CRP or depressive symptoms, with some studies considering the effect of interval time (37, 38). We recalculated cumulative effect of repeated episodes of CRP or depressive symptoms as follows: ( $\text{Cumulative CRP= }\left(crp_{11}+crp_{15}\right)/2\times time_{11-15}$ , $\text{Cumulative depressive symptoms= }\left(CESD-10_{11}+\;CESD-10_{13}\right)/2\times time_{11-13}$ ). We also duplicated the multivariate logistic regression model analyses in Sensitivity Analysis 5.

Data analyses were conducted using SAS 9.4 (SAS Institute, Inc) and STATA 16.0 (StataCorp LLC) software programs, with statistical significance set at P< 0.05 (two-tailed) for all analyses.

## 3 Results

### 3.1 Analysis 1: the bidirectional associations based on only single determination of CRP or depressive symptoms

Of the 4,961 individuals included in the analyses, the mean age was 58.2 ± 8.4 years and 46.70% of them were males. Furthermore, 629 (12.68%) individuals had elevated CRP levels, while 1,805 (36.38%) individuals experienced elevated depressive symptoms at baseline. The sample characteristics, categorized by varying levels of CRP and depressive symptoms at baseline, are presented in **Supplementary Table S1**.

**Table 1** illustrates the associations between elevated CRP levels at baseline and elevated depressive symptoms at follow-up, as well as between elevated depressive symptoms at baseline and elevated CRP at follow-up. Non-significant results were observed in both directions. Furthermore, in sex-stratified analyses, the lack of a significant relationship remained unchanged.

**Table 1:** Bi-directional associations based on only single determination of exposure between depressive symptoms and CRP at baseline (2011y) and follow-up (2015y).

Exposure (2011y)	Outcome (2015y)		Model 1 OR (95%CI)	Model 2 OR (95%CI)	Model 3 OR (95%CI)	Model 4 OR (95%CI)	Model 5 OR (95%CI)
			Elevated CRP: ≥3 mg/L				
**CRP**	**Depressive symptoms**	**Total**	1.05 (0.87,1.27)	1.10 (0.91,1.33)	1.10 (0.91,1.34)	1.13 (0.93,1.37)	1.12 (0.92,1.37)
		**Male**	1.03 (0.78,1.38)	1.05 (0.79,1,41)	1.05 (0.78,1.40)	1.07 (0.80,1.43)	1.06 (0.79,1.42)
		**Female**	1.10 (0.85,1.43)	1.12 (0.86,1.45)	1.13 (0.87,1.47)	1.14 (0.88,1.49)	1.14 (0.88,1.48)
			Elevated depressive symptoms: CESD-10 ≥10				
**Depressive symptoms**	**CRP**	**Total**	1.02 (0.87,1.19)	1.01 (0.86,1.18)	0.98 (0.84,1.16)	1.02 (0.86,1.20)	1.00 (0.84,1.18)
		**Male**	1.02 (0.81,1.30)	0.99 (0.77,1.26)	0.96 (0.76,1.23)	1.00 (0.78,1.28)	0.99 (0.77,1.28)
		**Female**	1.01 (0.82,1.24)	1.03 (0.83,1.28)	1.01 (0.81,1.25)	1.04 (0.84,1.30)	1.01 (0.81,1.27)

CRP, C-reactive protein; CI, confidence interval; OR, odds ratio.

Reference defined as having low CRP:<3 mg/L and low depressive symptoms: CESD-10<10.

Model 1 are adjusted for baseline outcome.

Model 2 are Model 1 plus adjustment for socio-demographic characteristics (age, sex, residential area, education attainment, marital status).

Model 3 are Model 2 plus adjustment for health related behaviors (tobacco smoking status, alcohol consumption, social activity).

Model 4 are Model 3 plus adjustment for metabolic measures (BMI, HDL, triglycerides).

Model 5 are Model 4 plus adjustment for health status (hypertension, diabetes mellitus, cancer, CVD, arthritic, asthma at baseline).

**Table 1** Bidirectional associations based on a single determination of CRP or depressive symptoms between depressive symptoms and CRP.

### 3.2 Analysis 2: the bidirectional associations based on two successive determinations of CRP or depressive symptoms

#### 3.2.1 Analysis 2.1: Cumulative effect of sustained CRP elevations on depressive symptoms

Of the 4,107 individuals included in analyses, 3,090 individuals exhibited consistently low CRP levels, while 204 individuals had consistently elevated CRP over two successive determinations. **Supplementary Table S2** presents the sample characteristics related to varying occasions of CRP elevations over two successive determinations. Multivariate logistic regression models summarized the odds of developing depressive symptoms after 3 years (2018y) in **Table 2**.

**Table 2:** Odds of developing depressive symptoms after 3 years (2018y) among older adults based on the cumulative effect of sustained CRP elevations over two successive determinations(2011y,2015y).

	Occasions	Odds of developing depressive symptoms after 3 years				
		Model 1	Model 2	Model 3	Model 4	Model 5
		OR (95%CI)	OR (95%CI)	OR (95%CI)	OR (95%CI)	OR (95%CI)
Total (N=4107)	zero (n=3090)	1.00 (reference)	1.00 (reference)	1.00 (reference)	1.00 (reference)	1.00 (reference)
	one(n=813)	0.99 (0.84, 1.18)	1.00 (0.84, 1.19)	1.00 (0.84,1.19)	1.00 (0.84, 1.19)	1.00 (0.84, 1.19)
	two(n=204)	**1.54 (1.14, 2.09)**	**1.58 (1.16, 2.14)**	**1.57 (1.16,2.14)**	**1.62 (1.18, 2.21)**	**1.58 (1.15, 2.17)**
Male (N=1898)	zero (n=1432)	1.00 (reference)	1.00 (reference)	1.00 (reference)	1.00 (reference)	1.00 (reference)
	one(n=380)	0.94 (0.72, 1.22)	0.92 (0.71, 1.20)	0.92 (0.71,1.20)	0.92 (0.71, 1.21)	0.91 (0.69, 1.18)
	two(n=86)	**1.85 (1.15, 2.98)**	**1.91 (1.19, 3.07)**	**1.87 (1.16,3.00)**	**1.95(1.19, 3.17)**	**1.87 (1.15, 3.06)**
Female (N=2209)	zero (n=1658)	1.00 (reference)	1.00 (reference)	1.00 (reference)	1.00 (reference)	1.00 (reference)
	one(n=433)	1.05 (0.84, 1.32)	1.07 (0.85, 1.35)	1.07 (0.85,1.35)	1.07 (0.85, 1.35)	1.07 (0.85, 1.36)
	two(n=118)	1.35 (0.91, 2.01)	1.40 (0.94, 2.10)	1.42 (0.95,2.13)	1.46 (0.97, 2.19)	1.45 (0.96, 2.20)

CRP, C-reactive protein; CI, confidence interval; OR, odds ratio.

The number of occasions inflamed considers having CRP ≥3mg/L at either none of, one of or both of 2011y and 2015y.

Outcome defined as having depressive symptoms (CESD-10≥10) vs not having depressive Symptoms (CESD-10<10) in 2018y.

Model 1 are adjusted for baseline depression.

Model 2 are Model 1 plus adjustment for socio-demographic characteristics (age, sex, residential area, education attainment, marital status).

Model 3 are Model 2 plus adjustment for health related behaviors (tobacco smoking status, alcohol consumption, social activity).

Model 4 are Model 3 plus adjustment for metabolic measures (BMI, HDL, triglycerides).

Model 5 are Model 4 plus adjustment for health status (hypertension, diabetes mellitus, cancer, CVD, arthritic, asthma at baseline).The meaning of the bold value represents statistical significance of the calculated OR value.

After adjusting for baseline depressive symptoms, middle-aged and older adults with cumulative effects of CRP elevations on one occasion (1 vs. 0) exhibited non-significant odds of developing depressive symptoms (OR=0.99; 95% CI: 0.84 to 1.18), while those with cumulative effects of sustained CRP elevations on two occasions (2 vs. 0) showed significantly higher odds (OR=1.55; 95% CI: 1.14 to 2.09). Even after adjusting for the four sets of covariates, the elevated excess risk persisted among those with cumulative effects of sustained CRP elevations on two occasions (OR=1.58; 95% CI: 1.15 to 2.17).

In analyses stratified by gender, adjusting for baseline depressive symptoms, males with cumulative effects of sustained CRP elevations on two occasions(2 vs. 0) exhibited significantly heightened likelihood of experiencing symptoms of depression (OR=1.85; 95% CI: 1.15 to 2.98), while females with cumulative CRP on 2 vs. 0 occasions showed non-significantly increased odds (OR=1.35; 95% CI: 0.91 to 2.01). In males, these elevated odds persisted even after adjusting for all measured covariates (OR = 1.87, 95% CI = 1.15, 3.06).

**Table 2** Odds of developing depressive symptoms after 3 years among older adults based on the cumulative effect of sustained CRP elevations over two successive determinations.

#### 3.2.2 Analysis 2.2: Cumulative effect of repeated episodes of depression on CRP

Of the 4,804 individuals included in analyses, 2,493 individuals exhibited consistently low depressive symptoms, while 926 individuals had consistently elevated depressive symptoms over two successive determinations. **Supplementary Table S3** presents the sample characteristics concerning various occasions of repeated episodes of depression over two successive determinations. Multivariate logistic regression models summarized the odds of developing elevated CRP level after 2 years (2015y) among middle-aged and older adults, based on cumulative effect of repeated episodes of depression over two successive determinations (2011y, 2013y), in **Table 3**.

**Table 3:** Odds of developing inflammation after 2 years (2015y) among older adults based on the cumulative effect of repeated episodes of depression over two successive determinations (2011y,2013y).

	Occasions	Odds of developing inflammation after 2 years				
		Model 1	Model 2	Model 3	Model 4	Model 5
		OR (95%CI)	OR (95%CI)	OR (95%CI)	OR (95%CI)	OR (95%CI)
Total (N=4804)	zero (n=2493)	1.00 (reference)	1.00 (reference)	1.00 (reference)	1.00 (reference)	1.00 (reference)
	one (n=1385)	1.15 (0.97,1.38)	1.15 (0.96,1.37)	1.13 (0.95,1.36)	1.16 (0.97,1.39)	1.14 (0.95,1.37)
	two (n=926)	**1.27 (1.05,1.55)**	**1.24 (1.04,1.56)**	**1.24 (1.01,1.53)**	**1.30 (1.06,1.60)**	**1.26 (1.02,1.56)**
Male (N=2246)	zero (n=1352)	1.00 (reference)	1.00 (reference)	1.00 (reference)	1.00 (reference)	1.00 (reference)
	one (n=594)	0.98 (0.75,1.27)	0.94 (0.72,1.23)	0.94 (0.72,1.23)	0.96 (0.73,1.26)	0.96 (0.73,1.26)
	two (n=300)	1.33 (0.97,1.82)	1.26 (0.91,1.73)	1.23 (0.89,1.70)	1.29 (0.93,1.79)	1.27 (0.90,1.78)
Female (N=2558)	zero (n=1141)	1.00 (reference)	1.00 (reference)	1.00 (reference)	1.00 (reference)	1.00 (reference)
	one (n=791)	**1.31 (1.02,1.67)**	**1.33 (1.04,1.70)**	**1.32 (1.03,1.69)**	**1.34 (1.04,1.72)**	**1.31 (1.02,1.69)**

CRP, C-reactive protein; CI, confidence interval; OR, odds ratio.

The number of occasions depressive symptoms considers having CESD-10≥10 at either none of, one of or both of 2011y and 2013y.

Outcome defined as having inflammation (CRP ≥3mg/L) vs not having inflammation (CRP <3mg/L) in 2015.

Model 1 are adjusted for baseline CRP.

Model 2 are Model 1 plus adjustment for socio-demographic characteristics (age, sex, residential area, education attainment, marital status).

Model 3 are Model 2 plus adjustment for health related behaviors (tobacco smoking status, alcohol consumption, social activity).

Model 4 are Model 3 plus adjustment for metabolic measures (BMI, HDL, triglycerides).

Model 5 are Model 4 plus adjustment for health status (hypertension, diabetes mellitus, cancer, CVD, arthritic, asthma at baseline).The meaning of the bold value represents statistical significance of the calculated OR value.

After adjusting for baseline CRP, individuals with cumulative effect of episodes of depression on one occasion(1 vs. 0) exhibited non-significant odds of developing later elevated CRP levels (OR=1.15; 95% CI: 0.97 to 1.38), whereas those with cumulative effect of repeated episodes of depression on two occasions(2 vs. 0) showed significantly higher odds (OR=1.27; 95% CI: 1.05 to 1.51). Even after adjusting for the four sets of covariates, the elevated excess risk persisted among those with cumulative effect of repeated episodes of depression on two occasions (OR=1.26; 95% CI: 1.02 to 1.56).

In gender-stratified analyses, after adjusting for baseline CRP, males with cumulative effect of repeated episodes of depression on two occasions (2 vs. 0) did not exhibit significantly increased odds of later increasing CRP levels (OR=1.27; 95% CI: 0.98 to 1.65). These non-significant results persisted even after adjusting for all measured covariates (OR=1.31; 95% CI: 0.99 to 1.74). In contrast, females with cumulative effect of episodes of depression on one occasion (1 vs. 0) showed significantly increased odds after adjusting for the four sets of covariates (OR=1.31; 95% CI: 1.02 to 1.69). Females with cumulative effect of repeated episodes of depression on two occasions (2 vs. 0) also demonstrated some significant results after adjusting for socio-demographic characteristics in Model 2 and metabolic measures in Model 4.

**Table 3** Odds of developing elevated CRP levels after 2 years among older adults based on the cumulative effect of repeated episodes of depression over two successive determinations.

### 3.3 Sensitivity analyses

To assess whether excluding participants with elevated CRP or depressive symptoms at baseline affected our results, we conducted the same three main analyses in a sub-cohort. In Sensitivity Analysis 1, we excluded 1805 participants with elevated depressive symptoms at baseline and 629 participants with elevated CRP levels at baseline to investigate the bidirectional associations based on a single assessment of CRP or depressive symptoms (**Supplementary Table S4**). After adjusting for all covariates, females exhibited significantly higher odds of developing depressive symptoms (OR=1.48; 95% CI: 1.05 to 2.09). One possible explanation is that a higher proportion of females in the main Analysis 1 had baseline elevated depressive symptoms (62.99%) compared to males (37.01%), as indicated in **Supplementary Table S1**. In addition, 2668 individuals included in Sensitivity Analysis 2 after excluding 1439 participants with existing elevated depressive symptoms at baseline. The odds of developing depressive symptoms after 3 years were broadly consistent with the main analyses (**Supplementary Table S5**). Furthermore, Sensitivity Analysis 3 included 4185 individuals, focusing on cumulative effect of repeated episodes of depression to CRP. Although the association did not show statistical significance, the OR estimates were > 1 for both the total population and women (**Supplementary Table S6**). Notably, individuals excluded from the primary analytical sample due to baseline elevated CRP levels already had higher CES-D scores at baseline compared to those individuals with low CRP at baseline, resulting in the absence of directional effects of depressive symptoms on subsequent CRP in these individuals.

Considering issues related to collinearity and the absence of CRP data in 2013y, we adjusted for the average of the two depression observations in Sensitivity Analysis 4 when examining the relationship between cumulative effect of sustained CRP elevations to depressive symptoms (**Supplementary Table S7**). We also accounted for the effect of interval time in the calculation of cumulative effect of repeated episodes of CRP or depressive symptoms in Sensitivity Analysis 5 (**Supplementary Table S8**). The results of the two sensitivity analyses mentioned above remained consistent with the main analyses.

## 4 Discussion

Our study suggested that there are bi-directional associations between depressive symptoms and CRP among Chinese middle-aged and older adults. Findings from the present study indicated a significantly positive relationship between cumulative effect of sustained CRP elevations and subsequent depressive symptoms, as well as between cumulative effect of repeated episodes of depression and later CRP.

The current study proposed the pathway from elevated CRP levels to later elevated depressive symptoms. In our study, we observed that cumulative effect of sustained CRP elevations was a better predictor of later depressive symptoms than the current CRP levels alone. Similar results were also reported in previous population-based studies (18, 19). This pathway has garnered support from a growing body of evidence in the field of depression pathophysiology. Morimoto et al. hypothesized that inflammatory processes play a role in the pathogenesis of geriatric depression in the context of medical and neurological illnesses (39). Kitaoka et al. demonstrated in animal models that depressive-like behavior is observed as a consequence of neuroinflammation in the medial prefrontal cortex, which is triggered by chronic stress and initiated by microglia (40). Furthermore, Abhay et al. noted that certain genes linked to immune and inflammatory pathways are correlated with human depression based on genome sequencing and proteomic analyses (41). However, certain population-based studies did not provide support for the pathway from CRP to depression (13, 14). The possible reason is that these previous studies primarily focused on single determination and did not consider the chronic nature of inflammation. This approach may not accurately capture the long-term pattern and could potentially result in sample misclassification.

Furthermore, we also proposed a pathway from elevated depressive symptoms to subsequently increased CRP levels. Our study revealed CRP levels were more accurately predicted by the cumulative effect of repeated episodes of depression rather than current depressive symptoms itself. Consistent with our findings, prior research indicated that the impact of cumulative depressive episodes on subsequent CRP levels increased and remained significant even after adjusting for various covariates (23). However, the conclusions of above-mentioned study were derived from American populations, and their applicability to the Chinese population needed to be examined. Our study complemented prior literature by investigating the association between depressive symptoms and later CRP levels in a large sample of Chinese middle-aged and older adults. Although less attention had been paid to this pathway from depression to later CRP, a possible mechanism behind it may lie within existing associations of depression and inflammation (20–22). In conclusion, by combining the results of CRP with later depressive symptoms, our study revealed that depressive symptoms and CRP levels are intertwined, mutually influencing each other.

Our study revealed “cumulative effects” in the bidirectional relationship between CRP and depressive symptoms among the Chinese population. Our findings are not contradictory to a recently published study utilizing the same dataset (CHARLS), which reported no bidirectional association between CRP and depressive symptoms (28). Both studies yield similar results regarding the single determination of CRP or depressive symptoms; however, our contribution lies in identifying the cumulative effect of multiple episodes that exerts a more pronounced influence on later CRP levels or depressive symptoms.

Recognizing the chronic and persistent nature of exposure factors for epidemiological research is essential. Single assessment of exposure may result in misclassification, as it fails to distinguish whether the individual’s exposure is recurring. By comparing our results in direction of depressive symptoms to CRP, females with elevated depressive symptoms on 1 vs. 0 occasions showed markedly association to later CRP in Analysis 2.2, whereas non-significance was found in Analysis 1. One possible reason for this discrepancy is that Analysis 1 may have included misclassifications. Individuals who had low depressive symptoms in 2011y but experienced elevated depressive symptoms in 2013y were categorized as having 0 occasion of exposure in Analysis 1, while as having 1 occasion of exposure in Analysis 2.2. Due to the limited number of visit points in CHARLS, the present study presented bidirectional associations by the number of assessment points at which exposures exceeded the threshold. In longitudinal studies with multiple observation points, we also recommend that methods akin to trajectory modeling can be employed to more accurately capture the dynamic and persistent changes in exposure.

We supported that there was no difference between males and females in the bidirectional relationship between CRP and depressive symptoms. While we observed statistically significant results for the cumulative effect of sustained CRP elevations on depressive symptoms in the male population, the point values of ORs for the other sex were all greater than 1. In addition, significant results were observed in the cumulative effect of repeated episodes of depression on CRP in the female population, but the point values of ORs for the other sex were all greater than 1. However, one study reported sustained (rather than transient) CRP elevations were associated with future depressive symptoms among women (18). But the study had a limited sample size, and we anticipate that with a larger sample size, there may be no discernible difference in results between males and females. Furthermore, a study that employed a similar approach also did not uncover strong evidence supporting differential associations between inflammation and depression among men and women (42). Further biological and population-based studies are needed to confirm this hypothesis.

Our study is the first to investigate the bidirectional associations between CRP and depressive symptoms, taking cumulative effect of repeated episodes of CRP or depressive symptoms into account, among middle-aged and older Chinese adults. We considered not only single determinations but also two successive determinations of CRP or depressive symptoms to comprehensively examine the bidirectional associations in a more appropriate manner. In addition, we followed a relatively large nationally representative cohort over a follow-up period of 8 years. There are some limitations to be noted despite these strengths. Firstly, due to 4 observation points for depressive symptoms but only 2 for CRP levels, the analyses of cumulative effect of repeated episodes of CRP or depressive symptoms in bidirectional associations had inconsistent time intervals. Consequently, it was challenging to rule out the influence of period-related confounding factors. Secondly, there is a possibility of selection bias because we excluded participants without baseline measures of depressive symptoms, CRP, and follow-up data from our analyses. Thirdly, we diagnosed depressive symptoms based on the CESD-10 questionnaire rather than relying on medical professionals, which may not accurately reflect clinically diagnosed episodes of depression. Finally, due to the restricted number of observation points in CHARLS, this study depicted the reciprocal connections between depression and inflammation. This makes it difficult to establish causality. To fully elucidate this relationship, we recommend that future investigations use extended longitudinal data with a temporal framework and adopt more rigorous causal modeling techniques.

## 5 Conclusion

Our study provides robust evidence supporting bidirectional associations between CRP and depressive symptoms, considering the cumulative effect of repeated episodes of CRP or depressive symptoms, among middle-aged and older Chinese adults. Overall, these findings of positive and bidirectional relationship between CRP and depressive symptoms add to a growing body of evidence examining the interplay between biological and psychological processes. These findings hold potential clinical significance for underscoring the promise of both anti-inflammatory and antidepressant approaches.

## Data availability statement

Publicly available datasets were analyzed in this study. This data can be found here: The datasets supporting the conclusions of this article are available publicly, http://charls.pku.edu.cn/.

## Ethics statement

The ethical approval of data collection was from the Biomedical Ethics Review Committee of Peking University (IRB00001052–11015). Every participant signed an informed consent before investigation, and their information were kept anonymous. All methods were performed in accordance with the relevant guidelines and regulations.

## Author contributions

NZ: Conceptualization, Data curation, Formal Analysis, Methodology, Visualization, Writing – original draft. LJ: Conceptualization, Data curation, Methodology, Writing – review & editing. MH: Data curation, Formal Analysis, Validation, Writing – review & editing. BZ: Conceptualization, Methodology, Writing – review & editing. YL: Validation, Writing – review & editing. QY: Methodology, Writing – review & editing. JH: Supervision, Writing – review & editing. CZ: Conceptualization, Formal Analysis, Methodology, Supervision, Validation, Writing – review & editing.
