# Supplementary material for: Bidirectional relationship between C-reactive protein and depressive symptoms considering cumulative effect among Chinese middle-aged and older adults
Source: Front Psychiatry. 2024 Jun 14;15:1319682. doi: 10.3389/fpsyt.2024.1319682 (PMC11211587; doi:10.3389/fpsyt.2024.1319682)
Supplement: Supplementary file 1 [file Table_1.docx]

Supplementary Material

# Supplementary Tables

**Table S1 Characteristics of participants of Analysis 1**

|  | **Low CRP**  **(<3.0 mg/L)**  **（n=4332）** | **Elevated CRP**  **(≥3.0 mg/L)**  **（n=629）** | **Low depressive symptoms**  **(CESD -10<10)**  **（n=3156）** | **Elevated depressive symptoms**  **(CESD-10≥10)**  **（n=1805）** | **Total**  **（N=4961）** |
| --- | --- | --- | --- | --- | --- |
| Baseline hs-crp(mg/l) , mean ± SD | 1.02±0.68 | 4.92±1.71 | 1.54±1.59 | 1.48±1.52 | 1.52±1.57 |
| Baseline depressive symptoms scores, mean ± SD | 8.25±6.22 | 8.50±6.20 | 4.37±2.75 | 15.11±4.44 | 8.28±6.22 |
| Elevated CRP ,n (%) | - | - | 396(12.55) | 233(12.91) | 629(12.68) |
| Elevated depressive symptoms ,n (%) | 1359(31.37) | 209(33.23) | - | - | 1568(31.61) |
| Age(years), mean ± SD | 57.96±8.37 | 60.01±8.42 | 57.82±8.31 | 58.91±8.53 | 58.22±8.40 |
| Sex, n (%) |  |  |  |  |  |
| Male | 2002(46.21) | 315(50.08) | 1649(52.25) | 668(37.01) | 2317(46.70) |
| Female | 2330(53.79) | 314(49.92) | 1507(47.75) | 1137(62.99) | 2644(53.30) |
| Residential area, n (%) |  |  |  |  |  |
| Rural | 3659(84.46) | 518(82.35) | 2777(81.84) | 1400(89.29) | 4177(84.20) |
| Urban | 673(15.54) | 111(17.65) | 616(18.16) | 168(10.71) | 784(15.80) |
| Educational attainment, n (%) |  |  |  |  |  |
| Illiterate | 1058(24.42) | 168(26.71) | 646(20.47) | 580(32.13) | 1226(24.71) |
| Elementary school | 1886(43.54) | 275(43.72) | 1327(42.05) | 834(46.20) | 2161(43.56) |
| Middle school | 939(21.68) | 127(20.19) | 785(24.87) | 281(15.57) | 1066(21.49) |
| High school or above | 449(10.36) | 59(9.38) | 398(12.61) | 110(6.09) | 508(10.24) |
| Marital status, n (%) |  |  |  |  |  |
| Married | 3937(90.88) | 562(89.35) | 2916(92.40) | 1583(87.70) | 4499(90.69) |
| Divorced | 21(0.48) | 5(0.79) | 11(0.35) | 15(0.83) | 26(0.52) |
| Widowed | 349(8.06) | 61(9.70) | 218(6.91) | 192(10.64) | 410(8.26) |
| Never married | 25(0.58) | 1(0.16) | 11(0.35) | 15(0.83) | 26(0.52) |
| Tobacco smoking status, n (%) |  |  |  |  |  |
| Never smoker | 2672(61.68) | 356(56.60) | 1848(58.56) | 1180(65.37) | 3028(61.04) |
| Former smoker | 341(7.87) | 69(10.97) | 267(8.46) | 143(7.92) | 410(8.26) |
| Current smoker | 1319(30.45) | 204(32.43) | 1041(32.98) | 482(26.70) | 1523(30.70) |
| Alcohol consumption, n (%) |  |  |  |  |  |
| Never drinker | 2521(58.19) | 367(58.35) | 1781(56.43) | 1107(61.33) | 2888(58.21) |
| Former drinker | 337(7.78) | 60(9.54) | 208(6.59) | 189(10.47) | 397(8.00) |
| Current drinker | 1474(34.03) | 202(32.11) | 1167(36.98) | 509(28.20) | 1676(33.78) |
| Social activity, n (%) |  |  |  |  |  |
| 0 | 2119(48.92) | 291(46.26) | 1440(45.63) | 970(53.74) | 2410(48.58) |
| 1-2 | 990(22.85) | 139(22.10) | 713(22.59) | 416(23.05) | 1129(22.76) |
| ≥3 | 1223(28.23) | 199(31.64) | 1003(31.78) | 419(23.21) | 1422(28.66) |
| BMI, mean ± SD | 23.60±3.82 | 24.63±4.53 | 23.97±3.94 | 23.32±3.88 | 23.73±3.93 |
| Triglycerides(mg/dl) ,mean ± SD | 132.92±108.84 | 146.14±111.70 | 136.56±111.48 | 131.15±105.28 | 134.59±109.29 |
| HDL-C (mg/dl) ,mean ± SD | 51.64±15.24 | 47.59±15.33 | 50.34±15.06 | 52.51±15.64 | 51.13±15.31 |
| Hypertension, n (%) | 2384(55.03) | 425(67.57) | 1818(57.60) | 991(54.90) | 2809(56.62) |
| diabetes mellitus, n (%) | 606(13.99) | 126(20.03) | 453(14.35) | 279(15.46) | 732(14.76) |
| Cancer, n (%) | 33(0.76) | 9(1.43) | 21(0.67) | 21(1.16) | 42(0.85) |
| CVD, n (%) | 467(10.78) | 85(13.51) | 285(9.03) | 267(14.79) | 552(11.13) |
| Arthritic, n (%) | 1470(33.93) | 247(39.27) | 879(27.85) | 838(46.43) | 1717(34.61) |
| Asthma, n (%) | 140(3.23) | 29(4.61) | 66(2.09) | 103(5.71) | 169(3.41) |

**Table S2 Characteristics of participants of Analysis 2.1**

|  | **Elevated CRP on zero occasion**  **（n=3090）** | **Elevated CRP on one occasion**  **（n=813）** | **Elevated CRP on two occasions**  **（n=204）** |
| --- | --- | --- | --- |
| Baseline hs-crp(mg/l) , mean ± SD | 0.97±0.65 | 2.70±2.14 | 5.00±1.68 |
| Baseline depressive symptoms scores,  mean ± SD | 8.01±6.11 | 8.24±6.20 | 8.11±6.24 |
| High CRP ,n (%) | - | - | - |
| Elevated depressive symptoms ,n (%) | 924(29.90) | 256(31.49) | 61(29.90) |
| Age(years), mean ± SD | 57.13±7.84 | 58.16±8.00 | 58.93±7.68 |
| Sex, n (%) |  |  |  |
| Male | 1432(46.34) | 380(46.74) | 86(42.16) |
| Female | 1658(53.66) | 433(53.26) | 118(57.84) |
| Residential area, n (%) |  |  |  |
| Rural | 2649(85.73) | 685(84.26) | 155(75.98) |
| Urban | 441(14.27) | 128(15.74) | 49(24.02) |
| Educational attainment, n (%) |  |  |  |
| Illiterate | 708(22.91) | 182(22.39) | 51(25.00) |
| Elementary school | 1295(41.91) | 381(46.86) | 91(44.61) |
| Middle school | 749(24.24) | 173(21.28) | 41(20.10) |
| High school or above | 338(10.94) | 77(9.47) | 21(10.29) |
| Marital status, n (%) |  |  |  |
| Married | 2846(92.10) | 740(91.02) | 184(90.20) |
| Divorced | 16(0.52) | 3(0.37) | 2(0.98) |
| Widowed | 217(7.02) | 66(8.12) | 18(8.82) |
| Never married | 11(0.36) | 4(0.49) | 0(0) |
| Tobacco smoking status, n (%) |  |  |  |
| Never smoker | 1909(61.78) | 488(60.02) | 123(60.29) |
| Former smoker | 234(7.57) | 78(9.59) | 23(11.27) |
| Current smoker | 947(30.65) | 247(30.38) | 58(28.43) |
| Alcohol consumption, n (%) |  |  |  |
| Never drinker | 1807(58.48) | 475(58.43) | 131(64.22) |
| Former drinker | 220(7.12) | 64(7.87) | 26(12.75) |
| Current drinker | 1063(34.40) | 274(33.70) | 47(23.04) |
| Social activity, n (%) |  |  |  |
| 0 | 1506(48.74) | 393(48.34) | 85(41.67) |
| 1-2 | 698(22.59) | 192(23.62) | 47(23.04) |
| ≥3 | 886(28.67) | 228(28.04) | 72(35.29) |
| BMI, mean ± SD | 23.60±3.75 | 24.06±4.05 | 26.41±5.27 |
| Triglycerides(mg/dl) ,mean ± SD | 125.57±84.10 | 148.15±105.26 | 181.29±152.56 |
| HDL-C (mg/dl) ,mean ± SD | 51.94±14.96 | 48.82±14.96 | 43.51±14.15 |
| Hypertension, n (%) | 1621(52.46) | 512(62.98) | 152(74.51) |
| diabetes mellitus, n (%) | 383(12.39) | 143(17.59) | 55(26.96) |
| Cancer, n (%) | 27(0.87) | 9(1.11) | 1(0.49) |
| CVD, n (%) | 320(10.36) | 92(11.32) | 30(14.71) |
| Arthritic, n (%) | 1015(32.85) | 295(36.29) | 99(48.53) |
| Asthma, n (%) | 86(2.78) | 35(4.31) | 7(3.43) |

**Table S3 Characteristics of participants of Analysis 2.2**

| **N=4804** | **Elevated depressive symptoms on zero occasions**  **（N=2493）** | **Elevated depressive symptoms on one occasion**  **（N=1385）** | **Elevated depressive symptoms on two occasions**  **（N=926）** |
| --- | --- | --- | --- |
| Baseline hs-crp(mg/l) , mean ± SD | 1.55±1.60 | 1.49±1.55 | 1.48±1.48 |
| Baseline depressive symptoms scores, mean ± SD | 4.16±2.76 | 10.22±5.32 | 16.13±4.67 |
| High CRP ,n (%) | 330(13.24) | 172(12.42) | 117(12.63) |
| Elevated depressive symptoms ,n (%) | - | - | - |
| Age(years), mean ± SD | 58.15±8.40 | 58.86±8.61 | 58.62±8.30 |
| Sex, n (%) |  |  |  |
| Male | 1352(54.23) | 594(42.89) | 300(32.40) |
| Female | 1141(45.77) | 791(57.11) | 626(67.60) |
| Residential area, n (%) |  |  |  |
| Rural | 2255(81.14) | 1133(88.45) | 670(90.05) |
| Urban | 524(18.86) | 148(11.55) | 74(9.95) |
| Educational attainment, n (%) |  |  |  |
| Illiterate | 509(20.42) | 380(27.44) | 322(34.77) |
| Elementary school | 1011(40.55) | 640(46.21) | 421(45.46) |
| Middle school | 637(25.55) | 264(19.06) | 135(14.58) |
| High school or above | 336(13.48) | 101(7.29) | 48(5.18) |
| Marital status, n (%) |  |  |  |
| Married | 2312(92.74) | 1226(88.52) | 805(86.93) |
| Divorced | 8(0.32) | 7(0.51) | 7(0.76) |
| Widowed | 165(6.62) | 142(10.25) | 108(11.66) |
| Never married | 8(0.32) | 10(0.72) | 6(0.65) |
| Tobacco smoking status, n (%) |  |  |  |
| Never smoker | 1436(57.60) | 868(62.67) | 628(67.82) |
| Former smoker | 230(9.23) | 109(7.87) | 64(6.91) |
| Current smoker | 827(33.17) | 408(29.46) | 234(25.27) |
| Alcohol consumption, n (%) |  |  |  |
| Never drinker | 1376(55.19) | 824(59.49) | 604(65.23) |
| Former drinker | 174(6.98) | 127(9.17) | 95(10.26) |
| Current drinker | 943(37.83) | 434(31.34) | 227(24.51) |
| Social activity, n (%) |  |  |  |
| 0 | 1099(44.08) | 734(53.00) | 494(53.35) |
| 1-2 | 580(23.27) | 313(22.60) | 216(23.33) |
| ≥3 | 814(32.65) | 338(24.40) | 216(23.33) |
| BMI, mean ± SD | 23.97±3.80 | 23.65±4.25 | 23.11±3.87 |
| Triglycerides(mg/dl) ,mean ± SD | 134.49±91.65 | 130.31±93.93 | 130.56±93.53 |
| HDL-C (mg/dl) ,mean ± SD | 50.28±15.16 | 51.71±14.89 | 52.73±15.69 |
| Hypertension, n (%) | 1461(58.60) | 785(56.68) | 498(53.78) |
| diabetes mellitus, n (%) | 364(14.60) | 210(15.16) | 135(14.58) |
| Cancer, n (%) | 15(0.60) | 18(1.30) | 12(1.30) |
| CVD, n (%) | 225(9.03) | 152(10.97) | 168(18.14) |
| Arthritic, n (%) | 633(25.39) | 514(37.11) | 476(51.40) |
| Asthma, n (%) | 52(2.09) | 51(3.68) | 65(7.02) |

**Table S4 Sensitivity Analysis 1: Bi-directional associations based on only single determination of exposure between depressive symptoms and CRP at baseline (2011y) and follow-up (2015y)**

| **Exposure**  **(2011y)** | **Outcome**  **(2015y)** |  | **Model 1**  **OR (95%CI)** |  | | **Model 2**  **OR (95%CI)** |  | | **Model 3**  **OR (95%CI)** |  | **Model 4**  **OR (95%CI)** |  | **Model 5**  **OR (95%CI)** |
| --- | --- | --- | --- | --- | --- | --- | --- | --- | --- | --- | --- | --- | --- |
|  | | | **Elevated CRP: ≥3 mg/L（N=3156）** | | | | | | | | | | |
| **CRP** | **Depressive symptoms** | **Total** | 1.20(0.94,1.53) | |  | 1.24(0.97,1.59) |  | 1.27(0.99,1.63) | |  | **1.30(1.01,1.67)** |  | 1.29(0.99,1.66) |
|  |  | **Male** | 1.02(0.70,1.49) | |  | 0.99(0.67,1,47) |  | 0.99(0.67,1.48) | |  | 1.03(0.70,1.53) |  | 1.02(0.68,1.52) |
|  |  | **Female** | **1.41(1.01,1.95)** | |  | **1.46(1.05,2.04)** |  | **1.52(1.08,2.12)** | |  | **1.50(1.07,2.11)** |  | **1.48(1.05,2.09)** |
|  | | | **Elevated depressive symptoms: CESD-10 ≥10（N=4332）** | | | | | | | | | | |
| **Depressive symptoms** | **CRP** | **Total** | 0.98(0.82,1.16) | |  | 0.97(0.81,1.16) |  | 0.95(0.80,1.14) | |  | 0.98(0.82,1.18) |  | 0.98(0.81,1.18) |
|  |  | **Male** | 1.02(0.78,1.33) | |  | 0.96(0.72,1.26) |  | 0.94(0.71,1.25) | |  | 0.98(0.74,1.30) |  | 0.97(0.73,1.30) |
|  |  | **Female** | 0.96(0.76,1.20) | |  | 0.99(0.78,1.25) |  | 0.97(0.76,1.23) | |  | 0.99(0.78,1.27) |  | 0.99(0.77,1.28) |

Abbreviations: CRP, C-reactive protein; CI, confidence interval; OR, odds ratio.

Reference defined as having low CRP:＜3 mg/L and low depressive symptoms: CESD-10＜10.

Model 1 is null model.

Model 2 are Model 1 plus adjustment for socio-demographic characteristics (age, sex, residential area, education attainment, marital status).

Model 3 are Model 2 plus adjustment for health related behaviors (tobacco smoking status, alcohol consumption, social activity).

Model 4 are Model 3 plus adjustment for metabolic measures (BMI, HDL, triglycerides).

Model 5 are Model 4 plus adjustment for health status (hypertension, diabetes mellitus, cancer, CVD, arthritic, asthma at baseline).

**Table S5 Sensitivity Analysis 2: Odds of developing depressive symptoms after 3 years (2018y) among older adults based on the cumulative effect of sustained CRP elevations over two successive determinations(2011y,2015y)**

|  | **Occasions** | **Odds of developing depressive symptoms after 3 years** | | | | | | | | |
| --- | --- | --- | --- | --- | --- | --- | --- | --- | --- | --- |
|  |  | **Model 1** |  | **Model 2** |  | **Model 3** |  | **Model 4** |  | **Model 5** |
|  |  | **OR (95%CI)** |  | **OR (95%CI)** |  | **OR (95%CI)** |  | **OR (95%CI)** |  | **OR (95%CI)** |
| Total  (N=2668) | zero (n=2008) | 1.00 (reference) |  | 1.00 (reference) |  | 1.00 (reference) |  | 1.00 (reference) |  | 1.00 (reference) |
|  | one(n=525) | 0.96 (0.77, 1.19) |  | 0.96 (0.77, 1.19) |  | 0.95 (0.76,1.19) |  | 0.96(0.77, 1.20) |  | 0.96 (0.77, 1.20) |
|  | two(n=135) | **1.50 (1.01, 2.10)** |  | **1.46 (1.01, 2.12)** |  | **1.50 (1.03, 2.18)** |  | **1.53 (1.04, 2.24)** |  | **1.47 (1.00, 2.17)** |
| Male  (N=1279) | zero (n=1051) | 1.00 (reference) |  | 1.00 (reference) |  | 1.00 (reference) |  | 1.00 (reference) |  | 1.00 (reference) |
|  | one(n=268) | 0.81 (0.58, 1.14) |  | 0.82 (0.58, 1.15) |  | 0.81 (0.58, 1.14) |  | 0.81 (0.58, 1.15) |  | 0.81 (0.57, 1.14) |
|  | two(n=60) | **1.83 (1.16, 2.91)** |  | **2.06 (1.19, 3.59)** |  | **2.01 (1.14, 3.53)** |  | **2.06 (1.16, 3.66)** |  | **1.89 (1.05, 3.39)** |
| Female  (N=1289) | zero (n=957) | 1.00 (reference) |  | 1.00 (reference) |  | 1.00 (reference) |  | 1.00 (reference) |  | 1.00 (reference) |
|  | one(n=257) | 1.07 (0.80, 1.43) |  | 1.09 (0.81, 1.46) |  | 1.08 (0.80, 1.45) |  | 1.02 (0.72, 1.44) |  | 1.02 (0.72, 1.45) |
|  | two(n=75) | 1.10 (0.67, 1.79) |  | 1.17 (0.71, 1.92) |  | 1.24 (0.75, 2.05) |  | 1.09 (0.56, 2.11) |  | 1.07 (0.55, 2.08) |

Abbreviations: CRP, C-reactive protein; CI, confidence interval; OR, odds ratio.

The number of occasions inflamed considers having CRP ⩾3mg/L at either none of, one of or both of 2011y and 2015y.

Outcome defined as having depressive symptoms (CESD-10≥10) vs not having depressive Symptoms (CESD-10＜10) in 2018y.

Model 1 is null model.

Model 2 are Model 1 plus adjustment for socio-demographic characteristics (age, sex, residential area, education attainment, marital status).

Model 3 are Model 2 plus adjustment for health related behaviors (tobacco smoking status, alcohol consumption, social activity).

Model 4 are Model 3 plus adjustment for metabolic measures (BMI, HDL, triglycerides).

Model 5 are Model 4 plus adjustment for health status (hypertension, diabetes mellitus, cancer, CVD, arthritic, asthma at baseline).

**Table S6 Sensitivity Analysis 3: Odds of developing elevated CRP after 2 years (2015y) among older adults based on the the cumulative effect of repeated episodes of depression over two successive determinations(2011y,2013y)**

|  | **Occasions** | **Odds of developing inflammation after 2 years** | | | | | | | | |
| --- | --- | --- | --- | --- | --- | --- | --- | --- | --- | --- |
|  |  | **Model 1** |  | **Model 2** |  | **Model 3** |  | **Model 4** |  | **Model 5** |
|  |  | **OR (95%CI)** |  | **OR (95%CI)** |  | **OR (95%CI)** |  | **OR (95%CI)** |  | **OR (95%CI)** |
| Total  (N=4185) | zero (n=2163) | 1.00 (reference) |  | 1.00 (reference) |  | 1.00 (reference) |  | 1.00 (reference) |  | 1.00 (reference) |
|  | one(n=1213) | 1.08(0.89,1.32) |  | 1.07(0.88,1.31) |  | 1.06(0.87,1.30) |  | 1.08(0.88,1.32) |  | 1.08(0.88,1.32) |
|  | two(n=809) | 1.17(0.94,1.46) |  | 1.17(0.93,1.47) |  | 1.15(0.91,1.45) |  | 1.20(0.95,1.51) |  | 1.18(0.93,1.50) |
| Male  (N=1936) | zero (n=1168) | 1.00 (reference) |  | 1.00 (reference) |  | 1.00 (reference) |  | 1.00 (reference) |  | 1.00 (reference) |
|  | one(n=519) | 0.92(0.68,1.24) |  | 0.86(0.64,1.17) |  | 0.87(0.64,1.18) |  | 0.88(0.65,1.20) |  | 0.88(0.65,1.20) |
|  | two(n=249) | 1.28(0.89,1.83) |  | 1.15(0.80,1.67) |  | 1.15(0.79,1.67) |  | 1.20(0.82,1.75) |  | 1.18(0.80,1.75) |
| Female  (N=2249) | zero (n=995) | 1.00 (reference) |  | 1.00 (reference) |  | 1.00 (reference) |  | 1.00 (reference) |  | 1.00 (reference) |
|  | one(n=694) | 1.23(0.94,1.61) |  | 1.26(0.96,1.66) |  | 1.25(0.95,1.64) |  | 1.27(0.96,1.68) |  | 1.27(0.96,1.68) |
|  | two(n=560) | 1.15(0.86,1.54) |  | 1.23(0.91,1.65) |  | 1.21(0.90,1.63) |  | 1.26(0.93,1.70) |  | 1.27(0.93,1.73) |

Abbreviations: CRP, C-reactive protein; CI, confidence interval; OR, odds ratio.

The number of occasions depressive symptoms considers having CESD-10≥10 at either none of, one of or both of 2011 and 2013.

Outcome defined as having inflammation (CRP ⩾3mg/L) vs not having inflammation (CRP ＜3mg/L) in 2015.

Model 1 is null model.

Model 2 are Model 1 plus adjustment for socio-demographic characteristics (age, sex, residential area, education attainment, marital status).

Model 3 are Model 2 plus adjustment for health related behaviors (tobacco smoking status, alcohol consumption, social activity).

Model 4 are Model 3 plus adjustment for metabolic measures (BMI, HDL, triglycerides).

Model 5 are Model 4 plus adjustment for health status (hypertension, diabetes mellitus, cancer, CVD, arthritic, asthma at baseline).

**Table S7 Sensitivity Analysis 4: Odds of developing depressive symptoms after 3 years (2018y) among older adults based on the cumulative exposure to CRP (2011y,2015y)**

|  | **Occasions** | **Odds of developing depressive symptoms after 3 years** | | | | | | | | |
| --- | --- | --- | --- | --- | --- | --- | --- | --- | --- | --- |
|  |  | **Model 1** |  | **Model 2** |  | **Model 3** |  | **Model 4** |  | **Model 5** |
|  |  | **OR (95%CI)** |  | **OR (95%CI)** |  | **OR (95%CI)** |  | **OR (95%CI)** |  | **OR (95%CI)** |
| Total  (N=3496) | zero (n=2635) | 1.00 (reference) |  | 1.00 (reference) |  | 1.00 (reference) |  | 1.00 (reference) |  | 1.00 (reference) |
|  | one(n=692) | 0.93 (0.77, 1.13) |  | 0.93 (0.76, 1.13) |  | 0.93 (0.77, 1.13) |  | 0.93 (0.76, 1.14) |  | 0.93 (0.76, 1.13) |
|  | two(n=169) | **1.53 (1.07, 2.18)** |  | **1.58 (1.10, 2.26)** |  | **1.57 (1.10, 2.25)** |  | **1.60 (1.11, 2.30)** |  | **1.59 (1.10, 2.29)** |
| Male  (N=1653) | zero (n=1247) | 1.00 (reference) |  | 1.00 (reference) |  | 1.00 (reference) |  | 1.00 (reference) |  | 1.00 (reference) |
|  | one(n=329) | 0.88 (0.65, 1.18) |  | 0.86 (0.63, 1.16) |  | 0.86 (0.64, 1.17) |  | 0.86 (0.64, 1.17) |  | 0.85 (0.63, 1.16) |
|  | two(n=77) | **1.89 (1.12, 3.21)** |  | **2.00 (1.17, 3.40)** |  | **2.02 (1.18, 3.45)** |  | **2.07(1.20, 3.59)** |  | **2.00 (1.15, 3.48)** |
| Female  (N=1843) | zero (n=1388) | 1.00 (reference) |  | 1.00 (reference) |  | 1.00 (reference) |  | 1.00 (reference) |  | 1.00 (reference) |
|  | one(n=363) | 0.98 (0.76, 1.27) |  | 1.00 (0.77, 1.29) |  | 1.00 (0.77, 1.29) |  | 1.00 (0.77, 1.29) |  | 1.00 (0.76, 1.30) |
|  | two(n=92) | 1.28 (0.80, 2.06) |  | 1.33 (0.82, 2.14) |  | 1.33 (0.82, 2.14) |  | 1.36 (0.84, 2.22) |  | 1.41 (0.86, 2.31) |

Abbreviations: CRP, C-reactive protein; CI, confidence interval; OR, odds ratio.

The number of occasions inflamed considers having CRP ⩾3mg/L at either none of, one of or both of 2011 and 2015.

Outcome defined as having depressive symptoms (CESD-10≥10) vs not having depressive Symptoms (CESD-10＜10) in 2018.

Model 1 are adjusted the average=depression2011y+ depression2015y)/2.

Model 2 are Model 1 plus adjustment for socio-demographic characteristics (age, sex, residential area, education attainment, marital status).

Model 3 are Model 2 plus adjustment for health related behaviors (tobacco smoking status, alcohol consumption, social activity).

Model 4 are Model 3 plus adjustment for metabolic measures (BMI, HDL, triglycerides).

Model 5 are Model 4 plus adjustment for health status (hypertension, diabetes mellitus, cancer, CVD, arthritic, asthma at baseline).

**Table S8 Sensitivity Analysis 5: Bi-directional associations based on cumulative exposure between depressive symptoms and CRP**

|  |  | **Odds of developing depressive symptoms after 3 years** | | | | | | | | |
| --- | --- | --- | --- | --- | --- | --- | --- | --- | --- | --- |
|  |  | **Model 1** |  | **Model 2** |  | **Model 3** |  | **Model 4** |  | **Model 5** |
|  |  | **OR (95%CI)** |  | **OR (95%CI)** |  | **OR (95%CI)** |  | **OR (95%CI)** |  | **OR (95%CI)** |
| **Cumulative CRP to depression** | Total | 1.01 (0.99, 1.02) |  | 1.01 (0.99, 1.02) |  | 1.01 (0.99, 1.02) |  | 1.01 (0.99, 1.02) |  | 1.01 (0.99, 1.02) |
|  | Male | 1.01 (0.98, 1.02) |  | 1.01 (0.99, 1.03) |  | 1.01 (0.99, 1.03) |  | 1.01 (0.99, 1.03) |  | 1.01 (0.98, 1.03) |
|  | Female | 1.01 (0.99, 1.03) |  | 1.01 (0.99, 1.03) |  | 1.01 (0.99, 1.03) |  | 1.01 (0.99, 1.03) |  | 1.01 (0.99, 1.03) |
| **Cumulative depression to CRP** | Total | 1.01 (0.99, 1.02) |  | 1.00 (0.99, 1.01) |  | 1.00 (0.99, 1.01) |  | **1.01 (1.00, 1.01)** |  | **1.01 (1.00, 1.01)** |
|  | Male | 1.00 (0.99, 1.01) |  | 1.00 (0.99, 1.01) |  | 1.00 (0.99, 1.01) |  | 1.00 (0.99, 1.01) |  | 1.00 (0.99, 1.01) |
|  | Female | **1.01 (1.00, 1.01)** |  | **1.01 (1.00, 1.01)** |  | **1.01 (1.00, 1.01)** |  | **1.01 (1.00, 1.01)** |  | **1.01 (1.00, 1.02)** |

Abbreviations: CRP, C-reactive protein; CI, confidence interval; OR, odds ratio.

Cumulative CRP=


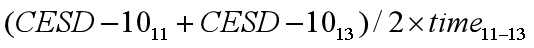
Cumulative depression=

Outcome defined as having depressive symptoms (CESD-10≥10) vs not having depressive Symptoms (CESD-10＜10) in 2018y.

Model 1 are adjusted for baseline outcome.

Model 2 are Model 1 plus adjustment for socio-demographic characteristics (age, sex, residential area, education attainment, marital status).

Model 3 are Model 2 plus adjustment for health related behaviors (tobacco smoking status, alcohol consumption, social activity).

Model 4 are Model 3 plus adjustment for metabolic measures (BMI, HDL, triglycerides).

Model 5 are Model 4 plus adjustment for health status (hypertension, diabetes mellitus, cancer, CVD, arthritic, asthma at baseline).
